# Editorial: Cancer cell adhesion, metastasis, and the immune response

**DOI:** 10.3389/fcell.2023.1347446

**Published:** 2023-12-12

**Authors:** Claudia Tanja Mierke

**Affiliations:** Faculty of Physics and Earth Systems Science, Peter Debye Institute of Soft Matter Physics, Biological Physics Division, Leipzig University, Leipzig, Germany

**Keywords:** epithelial-mesenchymal transition (EMT), cervical carcinoma, tumor microenvironment (TME), immunotherapy, ADAM8, cell mechanics, splicing factors, metastasis

The Research Topic (RT) is a comprehensive and highly relevant subject matter for the section of Cell Adhesion and Migration of Frontiers in Cell and Developmental Biology. The RT includes outstanding, high-impact manuscripts that are of interest to more than one research discipline. The RT covers two original research articles (Zhou et al. Luo et al.) and three review articles (Melo et al. Mierke; Zhou et al.) One of the original research articles deals with cervical cancer and its malignant progression with a focus on epithelial-mesenchymal transition (EMT) and autophagy (Zhou et al.) The other original research article comprises a pan-cancer approach to figure out whether splicing defects impacts cellular functions and cancer therapy outcome (Luo et al.) The first review article covers the impact of the tumor microenvironment on immunotherapies (Melo et al.) The second review article deals with posttranslational proteolytic cleavage induced by ADAM8 that alters directly or indirectly cancer cell behavior and characteristics (Mierke). The third review article emphasizes the role the α5β1 integrin, which binds the extracellular matrix protein fibronectin, in three specific cancer types: prostate cancer, renal cell carcinoma, and bladder cancer (Zhou et al.) These five articles show how broad the field of RT is and that molecular biological, biochemical, cell biological, medical and biophysical approaches, which belong to very different scientific disciplines, are expected to play a crucial role in understanding the complexity of cancer. Only the cooperation of these different areas and the analysis of different tumor types makes it possible to obtain a comprehensive picture of cancers and their malignant progression. In this way, similarities can be identified that would otherwise be lost or easily overlooked in the heterogeneity of tumors. In the following, the five outstanding articles will be described and their significance emphasized.

Zhou et al. investigated cervical cancers in their original research article with a focus on malignant progression through invasion and metastasis. Thereby the switch of the cancer cells’ phenotype from epithelial to mesenchymal, the EMT, plays a crucial role. They highlighted that autophagy-related genes (ARGs) function in EMT, whereby the process of autophagy fulfills two major functions in the cancer disease initiation and advancement (Chavez-Dominguez et al., 2020; Lim et al., 2021; Russell and Guan, 2022). There is no doubt that autophagy is an integral process that is triggered and stringently controlled under diverse cellular stress circumstances with the aim of reacting accurately to the continuously fluctuating microenvironment. It is meanwhile clear that autophagy has various functions in the pathogenesis of cancer. At the beginning of cancer development, autophagy suppresses cancer (Bao et al., 2020), while in the cancer progression phase it accelerates cancer (Galluzzi et al., 2015). The function of specific ARGs during EMT was not clear and therefore they explored the ARG signature in human cervical squamous cell carcinoma and human endocervical adenocarcinoma sequencing data basis. Thus, ATG5 has been revealed that alters survival rate in cervical cancer patients via Cox expression. Moreover, eight methylation sites within ATG5 correlate with overall survival of humans with cervical cancer and EMT level. Functional analysis revealed that silencing of ATG5 impaired migration and invasion. Consequently, the EMT is reversed to MET indicating that ATG5 controls the regulation of the EMT and fosters worse prognosis of cervical cancer. This study has utilized the cancer genome atlas.

Luo et al. used the cancer genome atlas to figure out in their original research article whether the splicing event on important splicing factor genes (SFG) is altered. Moreover, they even explored whether deregulated splicing can be found as a universal phenomenon in different types of cancer. Therefore, they studied the connection between mutations in SFG and clinical characteristics of cancer disease progression, integrity of the genomes, the immune responses to suppress the tumor and the reaction toward immunotherapy of typical cancer types. When SFG-wildtype cancers are compared with SFG-mutated cancers, the prognosis for patient survival is not good, the burden of the primary tumor is elevated, the amount of aneuploidy is raised, immunosuppressive profiles are increased, the level of tumor stemness is higher together with the increase in proliferation and the heterogeneities within the tumor are raised (Mierke et al., 2011b; Mierke et al., 2011a; Mierke, 2013). They reported that cancers bearing mutated SRG exhibit an elevated reaction rate to inhibitory substances toward immune checkpoints compared to SRG wildtype controls. On the individual cell level, SFG genes are linked to elevated cancer stemness, the proliferation level, PD-L1 expression, raised heterogeneity and amounts of aneuploidy. Consequently, the mutation in prominent SFGs couples to worse clinical prognosis, advancement of cancer, instability of the genome, immunosuppression to impair cancer growth and raised immunotherapy reactions to be present in pan-cancer. Finally, mutations in splicing factor genes are an adverse prognostic feature and a positive sign for immunotherapy reaction in cancer.

Immunotherapies have been investigated by Melo and colleagues and summarized in a review article by Melo and colleagues to alter the functional role of immune cells to combat cancer cells by using a variety of mechanisms Melo et al. Although these immunotherapies are effective in a wide range of cancer types, they are therapeutic for only a minority of patients. A key impediment to the effectiveness of immunotherapies lies in the immunosuppressive character of the tumor microenvironment (TME), which involves the stromal portion and the cancer immune system. In most solid cancers, the TME consists of a scarcely perfused vascular system due to pathological angiogenesis, such as leaky tumor blood vessels or cancer cell/endothelial cell vessel chimera that supply insufficient oxygen and inadequate nutrients. In addition, mechanical cues come into play (Mierke, 2014; Mierke, 2019; Pratt et al., 2020), such as the deregulated fibrosis encircling the malignancy, referred to as pathological desmoplasia, squeezes the tumor’s blood vessels tighter and restricts blood flow. TME must be normalized (closure of vascular leakages and function of cancerassociated fibroblasts in decompressing vessels), which constitutes a clinically proven strategy based on mathematical remodeling models (Stylianopoulos et al., 2018). The authors present in their review article the actual concepts and advancements in the partly revealed TME normalization process. Subsequently, findings on immunotherapy-induced TME normalization are presented and thoughts on the potential integration of vascular normalization in immunotherapies are considered. Finally, the authors conclude that understanding of TME can lead to a full normalization, which consequently improves the outcome of immunotherapies.

The review article by Mierke deals with the posttranslational proteolytic cleavage that denotes a unique and irreversible process regulating the functionality and half-life of multiple proteins. The review emphases one family member of the family of A disintegrin and metalloproteases (ADAMs), ADAM8. ADAM8 has attracted scrutiny in the regulation of diseases such as neurogenerative disorders, immune function and cancer by mitigating the function of proteins in proximity to the extracellular membrane scaffold. Thus, ADAM8 contributes to the ectodomain shedding process, which modifies the turnover rate of a number of transmembrane proteins that play a role in cell adhesion and receptor signaling. The review article is timely and focusses on the connection between ADAM8 and cancer. The author reviews and discusses ADAM8’s shedding function, direct and indirect matrix break-down, impacts on cancer cell motility and transmigration, and its interference with matrix-embedded adjacent cells. The most likely mechanical implications of ADAM8 on cancer cells and their matrix surroundings are also highlighted and discussed. In brief, this review provides the recent progress on the substrates/ligands and functionality of ADAM8 in its novel cancer role and its perspective association with the mechanical characteristics of cells (Hayn et al., 2023) and explains the matrix mechanics modifying features. Consequently, a more in-depth knowledge of the regulatory processes controlling the expression, subcellular distribution and activity of ADAM8 is anticipated to identify suitable therapeutic targets for customized and subtle manipulation of its proteolytic activity in cancer evolution and metastasis.

Zhou et al. focus in their review article on urological tumors, such as prostate cancer, renal cell carcinoma, and bladder cancer that are responsible for the growing incidents of malignant tumors. Metastasis of urological tumors to remote organs is the leading cause of cancer death, even though the mechanisms underpinning metastasis are not yet completely clear. However, it seems to be clear that the fibronectin receptor integrin α5β1 contributes to remote metastasis (Mierke et al., 2011b; Hou et al., 2020; Pantano et al., 2021). Moreover, this integrin is a widely recognized critical cancer facilitator as it interacts with various ligands, confers cancer adhesion, invasion and migration, and causes immune evasion. In this review article, the authors discuss the association and regulatory mechanisms of integrin α5β1 across these three cancer types. In particular, the clinical uses of integrin α5β1 in these cancers, particularly to combat treatment resistance, will be debated. Finally, the authors proposed the feasibility of integrin α5β1 as a candidate target for treatment and proposed new avenues for future investigation.

All five articles comprise the complexity of cancer cell adhesion, cancer metastasis and immune response from different angles by focusing on gene expression, mutation of genes, such as SFG, the impact of the TME and its remodeling by ADAM8, the integrin α5β1, EMT and its cooperation with autophagy. All this is related to extracellular mechanical cues of the microenvironment and intracellular mechanical characteristics of the cells. All articles clearly show how heterogeneous tumors can be and how divergent the individual tumor types can be. They thus challenge the concept of universal tumorigenesis and malignant progression as well as uniform alteration of tumor mechanics that reduce adhesion and ultimately enhance metastatic capacity. There are plenty of other interesting facts to look into, for example, whether the mechanics of organelles, such as the nucleus, also play a role, which in turn alters gene expression. It is necessary to study the different tumor types individually and when compared with others to find out whether further hallmarks of cancers can be identified.
